# Responding to clinical alarms in unfolding simulated clinical scenarios: auditory icons perform better than tonal alarms

**DOI:** 10.1016/j.bja.2024.12.047

**Published:** 2025-03-20

**Authors:** Judy Reed Edworthy, Natasha Talbot, Nicole Martin

**Affiliations:** School of Psychology, University of Plymouth, Plymouth, UK

**Keywords:** alarm fatigue, alarm signals, alarms, auditory icons, clinical alarms, clinical soundscape, monitoring, simulation

## Abstract

**Background:**

The international medical device safety standard IEC 60601-1-8 now recommends use of auditory icon alarms. Auditory icon alarms are alarms that act as metaphors for the problems that they signal. These are compared with traditional tonal alarms.

**Methods:**

Two sets of three auditory alarms were compared, one consisting of auditory icons and one consisting of variants of older tonal alarms. Volunteer participants were required to monitor three clinical scenarios each lasting 4 min with different problems (cardiac, oxygenation, and technical) occurring during each of these scenarios that triggered alarms multiple times. Participants were required to respond to those alarms while performing a separate vigilance task. Participants were taught the alarms before the tasks, learning the alarms either by the name of the problem (Hazard) or the position of the alarms in the sequence of events (Sequence).

**Results:**

Participants responded more accurately (*F*=23.48, *P*<0.05, eta^2^=0.32) and more quickly (*F*=20.357, *P*<0.001, eta^2^=0.51) to auditory icon alarms than to tonal alarms. This higher performance was not at the expense of performance on the vigilance task. The results showed no effect of learning the sounds as Hazards or as a Sequence.

**Conclusions:**

Auditory icons are more effective than tonal alarms. New alarms as indicated by the standard should be adopted by manufacturers wherever possible.

**Editor's key points**

• Alarm recognition and alarm fatigue in the clinical environment are challenges that are critically important to patient safety in hospital environments.
• This volunteer study compared auditory icons, which are real-world sounds that are easier to learn and are superior to traditional tonal alarms.
• Participants responded more accurately and more quickly to auditory icon alarms than to tonal alarms, without affecting concurrent performance on a vigilance task.
• Auditory icons, which are recommended by current industry standards, are more effective than tonal alarms, and should be adopted by device manufacturers wherever possible.

Clinical alarms alert clinicians to evolving situations that might otherwise be missed. Clinical alarms are so ubiquitous that ‘alarm fatigue’ is a known and much studied topic in clinical safety.^1^, ^2^, ^3^ Alarm fatigue is defined as ‘sensory overload when clinicians are exposed to an excessive number of alarms, which can result in desensitisation to alarms and missed alarms’.^4^ Alarm fatigue is a problem for a number of reasons, including the piecemeal way in which medical equipment is acquired and used in the day-to-day clinical environment, the multiple reasons why alarms trigger when there is no clinical problem, and issues around the limits of the technology used to produce auditory alarm signals coupled with a lack of understanding as to how humans (including clinicians) process and listen to sound.

IEC 60601-1-8 is a global standard concerned with medical device safety. Compliance with the standard is not mandated, but manufacturers are typically keen to show compliance with the standard because it contains many elements of good practice. The versions of IEC 60601-1-8 published in 2006 and 2012^5^ contain detailed specification of the auditory alarms that should be used for a set of clinical situations such as cardiovascular problems, oxygenation issues, and others. Testing showed that these alarms were very difficult to learn and retain.^6^^,^^7^ This is unsurprising as each of the eight alarms are identical in all ways except that they consisted of different tonal patterns (they were effectively different melodies). In all other ways, the eight alarms were identical; they possessed the same 3+2 pulse rhythm, in practice they possessed the same spectrum, and were played on the same octave range.

Increasing awareness of the conflicts between the intention behind the alarms and knowledge of the cognitive processing of sound, plus advances in the technology available for reproducing sound, led to calls for these alarm signals to be improved when the standard was updated, which happens every few years. This call included one of the original developers of the alarm sounds.^8^ In 2020 (IEC 2020), the alarm signals 60601-1-8 were updated in an extensive and well-publicised research programme. The alarms now recommended are ‘auditory icons’, which are real-world (or stylised real-world) sounds. These are much easier to learn and have been heavily tested before adoption (unlike previous alarm signals), and evidence for their superiority is available in the public domain.^9^, ^10^, ^11^, ^12^, ^13^, ^14^, ^15^, ^16^ These new alarms remove many of the problems associated with the use of alarms and alarm fatigue, though their uptake appears slow.

Tonal alarms or auditory icons are not the only alarms available; other types of alarms, in particular voice alarms and ‘spearcons’, which are shorter, speeded-up voice signals, have been shown to work well as alarms and can outperform even auditory icons.^17^, ^18^, ^19^, ^20^, ^21^ The relevant standards committees did not consider speech alarms as a viable option during the update of the standard for a number of reasons. These include the implications of mandating specific languages, probably English, in a global standard, the technical challenges of reproducing speech at the time that the standard was updated, and the implications of allowing alarms to be easily decodable by everyone, including patients and visitors. We focus on comparing the tonal alarms with auditory icons because these two types of alarm have special status in relation to the standard, and the implications for device manufacturers wishing to comply with the standard.

An unwell patient with monitoring devices attached is likely to generate a range of different alarms, from a ventilator indicating a breathing issue or technical issues associated with the ventilator, an oximeter indicating a blood–oxygen issue (SpO_2_), or electrocardiography indicating a cardiac issue. In practice, each of these issues or events is signalled by a single auditory alarm, the IEC 2006/2012 general alarm. This way of using alarms does not provide much information to the clinician about the developing clinical scenario. For example, a patient might be suffering a breathing issue that triggers a respiration rate alarm. This might be followed by a decreasing SpO_2_ causing an oxygenation alarm, which might then also develop into a cardiovascular issue, generating a further alarm. Most alarms are set up to re-signal after a minute or so. Thus on hearing an alarm signal, the clinician needs to first work out what event is signalling, and then on the occurrence of a second alarm, whether this is the first event re-signalling, or a second event. This can often escalate to the third and fourth event, compounding the general problem of alarm discrimination.

A solution to this alarm problem (and indeed one that the standard already allows for) is to use different alarm signals for each of the events. The developing event could be signalled by three separate and different alarms rather than the same signal. In this case the device would be set up to play specific alarm signals when a specific issue occurs. For this to be effective, the user needs to know which alarm sound is associated with which clinical event. Another way to signal that there is more than one event is to use adaptations of a single alarm to indicate whether the event is the first, second, or third alarm and beyond. In this study, we compared two sets of three alarm signals in a scenario of this type. One set is based on the auditory icons now proposed in the standard. The other set is based on the general alarm from previous versions of IEC 60601-1-8 but with adaptations so that there are three versions of the alarm intended to indicate three different positions of the event in the alarm sequence. We also tested two ways of labelling the alarm signals. Some participants learned the signals according to the hazards themselves (respiration, SpO_2_, or technical problem, named ‘Hazard’), whereas others learned them as the alarms associated with problems 1, 2, and 3 in sequence as they unfold (named ‘Sequence’). Participants responded to the alarms while performing a vigilance task known as the 2-back task, which we use as a continuous monitoring task as a simulation of clinical attention. This task requires participants to constantly monitor a screen on which letters appear singly for a few seconds, and to report when they see a letter that occurred two letters ago. This task, known as the N-back task where N is the number of letters between the first and second letter to be detected, is heavily used in cognitive psychology, where it has been used to address applied issues in aging, depression, and memory capacity.^22^, ^23^, ^24^, ^25^ It thus has significant provenance and validity as a task that taps into vigilance, memory, and other cognitive processes which would come into play when a clinician is performing an attention-demanding task that might be interrupted by alarms.

## Methods

Ethical clearance was granted by the Ethics Committee of the Faculty of Health, University of Plymouth, Plymouth, UK (November 2023).

A total of 83 volunteers participated in the study (eight male, 75 female, mean age 20.0 [range 18–35] yr). Participants were recruited from the University of Plymouth experimental participation system. Experiments were conducted online via a computer programme. The design of the experiment was 2×2 (sound type: Auditory icons; Tonal alarms) × (labelling: ‘Hazard’, H; ‘Sequence’, S), in a between-subjects design. Each participant completed one of the four conditions.

### Alarm sounds

Two sets of three alarms were used. The three alarms were used to represent either three clinical hazards: SpO_2_, respiratory rate, and a technical problem (Hazard); or the order in which alarms occurred: First, Second, and Third (Sequence). One set was three of the auditory icon alarm signals taken from the updated alarm sets associated with IEC 60601-1-8.^5^ These were the ventilation, oxygen, and power failure alarms. The other set was three variants of the 2006/2012^5^ general alarm from previous versions of the same standard, which are tonal alarms. Each consisted of a five-tone repeated pattern. The first-level alarm was represented by the existing general alarm, the second level by a similar sound with the pitch rise of one and then two tones in the last two pulses, and the third level started on a tone higher and rose in two steps of a musical third each for both of the last two tones. The whole alarm sound was also played slightly faster (10% faster than the first- and second-level alarms). The alarms are specified in Table 1. Both sets of alarms were learned using both sets of labels by different participants. Thus a quarter of the participants learned the auditory icon alarms as Hazard (‘respiration rate’, etc.), a quarter learned them as Sequences (‘First’, etc.), a quarter learned the tonal alarms as Hazards (‘respiration rate’, etc.), and a quarter learned them as Sequences (‘First’, etc.).

**Table 1:** Alarms used in the study. The auditory icons are described according to their meaning, the tonal alarms are described in terms of their tonal sequence where C4 is middle C. H, hazard; RR, respiratory rate; S, sequence; SpO_2,_ blood–oxygen issue; Tech, technical problem.

Alarm meaning	Auditory icon	Tonal alarm
First (S)/RR (H)	Human breathing in followed by breathing out lasting 3 s	D4-D4-D4-D4-D4 in 3 plus 2 pulse sequence, repeated, lasting 3.35 s
Second (S)/SpO_2_ (H)	Short pitched pulses bubbling lasting 2.1 s	D4-D4-D4-E4-Fsharp in 3 plus 2 sequence, repeated, lasting 3.35 s
Third (S)/Tech (H)	Power saw pulled on followed failing to start last 1.8 s	Eb4-Eb4-Eb4-G4-Bb4 in 3 plus 2 sequence, repeated, lasting 3.1 s

### Alarm timelines

Participants performed three separate alarm monitoring tasks with different timelines. The first timeline was designed to simulate how a clinical event might unfold in real time, with the first, then a second, and later a third problem being signalled, but with each alarm also being repeated after 1 min. The first timeline is shown in Table 2. The second timeline is the exact reverse of timeline 1, included for the purposes of experimental control. The third timeline was designed as a hybrid of the first two timelines, maintaining the condition that each alarm sounds again each successive minute after its initial sounding.

**Table 2:** Timeline of the first scenario. Participants learned the alarms either by Hazard or by Sequence. RR, respiratory rate; SpO_2,_ blood–oxygen issue; Tech, technical problem.

Time (s)	Hazard	Sequence
0	RR	First
50	SpO_2_	Second
60	RR	First
1.50	SpO_2_	Second
2.00	RR	First
2.30	Tech	Third
2.50	SpO_2_	Second
3.00	RR	First
3.10	Tech	Third
3.30	Tech	Third
3.50	SpO_2_	Second
4.00	RR	First

### Procedure

**Fig 1:**
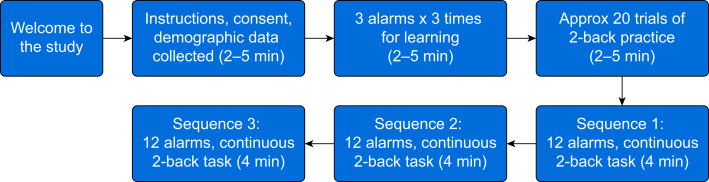
Timeline of study.

The complete timeline of the experiment is shown in Fig 1. Each participant logged into the programme site, consent was given, demographic data were collected, and a segment of music was heard in order for participants to set their computer volume and headphones at a reasonable listening level. They were briefed that they would be presented with three clinical auditory alarms which they should try to learn. They were told that they would carry out an experiment where they would be performing a continuous task (a 2-back task) while waiting for alarms to occur. When those alarms occurred, they were required to indicate which of the alarms they had heard by clicking on a box on screen. They were told that there would be three scenarios, each of which lasted ∼5 min. They then heard each of the alarms three times and were asked to try to learn them. They were then given some practice at the 2-back task. Here, letters appeared at regular intervals on a screen and their task was to press the spacebar when the letter currently presented had occurred two letters previously. They then proceeded to perform the experimental tasks. In the first task, they were presented with the first alarm timeline in Table 2. They were given feedback as to whether they were correct or incorrect on both their responses to the alarms and to the 2-back task. After a brief pause, they completed timeline 2, and after a further pause they completed timeline 3. After they had completed all three scenarios, they were debriefed, thanked for their participation, and were able to shut their browser.

### Statistical analysis

The statistical analysis plan was approved by the authors before data collection and analyses began. Analysis of the collected data was conducted using R Studio (Posit PBC).^26^

## Results

Values for the dependent variables correct response, reaction time, and N-back scores for the four conditions are shown in Table 3. Two-way Sound set × Label analyses of variance were calculated for accuracy, response time, and 2-back accuracy. The analysis for accuracy showed *F*=23.48 (*P*<0.01, df=1, eta^2^=0.32) for Sound set; *F*=1.40 (ns, df=1, eta^2^=0.08) for Labels; and *F*=0.23 (ns, df = 1,1, eta^2^=0.23) for the interaction. Thus responses were more accurate with the auditory icon alarms, and there were no other effects.

**Table 3:** Summary of results. Accuracy, mean number of correctly identified alarms; Icons, auditory icons; Tones, tonal alarms; RT (ms), mean response time in ms; 2-back accuracy, mean score on the 2-back task taking into account correct hits and misses, with a maximum score of 1. Data are presented as mean (sd).

		Accuracy	RT (ms)	2-back accuracy
Icons	Hazard	10.5 (1.05)	2970 (1018)	0.638 (0.154)
Icons	Sequence	10.9 (1.06)	2865 (1102)	0.485 (0.299)
Tones	Hazard	9.54 (1.04)	3371 (1277)	0.451 (0.210)
Tones	Sequence	9.68 (1.62)	3817 (1327)	0.519 (0.169)

For response time, analysis of variance (anova) showed a large main effect for the Sound set, with *F*=20.357 (*P*<0.001, df=1, eta^2^=0.51). It showed no main effect for Labels with *F*=0.917 (ns, df=1, eta^2^=0.11), and no interaction (*F*=3.232, ns, df = 1,1, eta^2^=0.20). anova for the 2-back task showed no main effect for the Sound set (*F*=2.587, ns, df=1, eta^2^=0.18), no main effect for Label (*F*=1.193, ns, df=1, eta^2^=0.12), and an interaction between Sound set and Label (*F*=5.372, *P*<0.05, df = 1,1, eta^2^=0.26).

## Discussion

We compared different types of alarm sounds and different ways of approaching their use in practice, and the influence of those two factors on volunteer participants' performance using those alarms and their labels. The sounds used were either auditory icons taken from the updated version of IEC 60601-1-8 (2020), or alarm sounds based on the older version of the general alarm of IEC 60601-1-8 (IEC 2006/2012). The latter trio of alarms possessed modifications to the old tonal alarms to enable participants to differentiate between the three levels representing first, second, and third hazards in a specific sequence, or to be used to differentiate between three different named hazards. The results for correct scores, reaction times, and scores on the 2-back task suggest that there was little variance in this study attributable to anything other than that owing to the auditory icon alarms performing better than the tonal alarms in producing greater accuracy and faster reaction times. This finding adds to the increasing body of evidence showing that auditory icon alarm sounds significantly outperform the tonal alarms frequently used in medical devices,^9^, ^10^, ^11^, ^12^, ^13^, ^14^, ^15^, ^16^ and can show significant benefits within a clinical monitoring scenario.^13^^,^^15^

Our data suggest that the labels ascribed to the sounds have little effect. Learning the sounds by clinical names (Hazard) or in order of occurrence (Sequence) appears to have no effect on task performance. This shows that there is little advantage in using workarounds such as using temporary order numbering as opposed to simply using specific problem names, if the alarms are easy to learn and discriminate. There are further experiments required to clarify this, but our data suggest that the problem lies with the alarm signals themselves. It will be interesting to discover what happens beyond three separate clinical problems. For auditory icons, we know that participants are able to learn and recognise the IEC auditory icons at 80% accuracy after only one exposure, increasing to 95–100% accuracy after a few exposures.^9^ We also know that the ability to learn and discriminate between two or more tonal alarms (the old IEC alarms) is slow and troublesome.^6^^,^^7^^,^^27^ We thus expect the difference between the auditory icons and the tonal alarms to become larger as the set of alarms to be discriminated between increases. Given that auditory icons are easy to learn, and that the standard now requires their use, there is little purpose in exploring the idea of sequencing clinical problems when the obvious and more straightforward solution is to assign specific alarm signals to specific functions, and to use the alarms in the way the standard advocates.

Another key reason for auditory icons being easy to learn is that they are very different from one another. This means that the listener has many cues available to tell them apart, making judgements of identity easier. Tonal alarms are more similar to one another, making them prone to masking and other audibility issues. Because they are complex harmonically, auditory icons are also easier to localise, which is often an important issue with clinical alarms.^9^^,^^10^

### The standard

IEC 60601-1-8 now stipulates that auditory icon alarms should be used in preference to the older tonal alarms, which is supported by significant evidence also contained within the standard. The are at least two important reasons for the superior performance. Firstly, auditory icons are more representative of their meaning, making them easier to learn and remember.^9^^,^^12^ Tonal alarms bear no relation to the functions that they represent and therefore require considerably more learning. With auditory icons and other metaphorical sounds such as ‘spearcons’,^20^^,^^21^ there is no need to fit words to tonal alarms in order to make them more memorable as the sounds are simply easy to learn.

Medical device manufacturers are at liberty to use whatever alarms they wish, so they can use other types of alarms such as voice warnings, which have also been shown to be effective and can outperform auditory icons.^17^, ^18^, ^19^, ^20^, ^21^ However, this would not be compliant with the standard. The standard does allow manufacturers to claim compliance if their own alarms outperform those specified in the standard, and metrics are provided in the standard in order to make this comparison. Therefore, if manufacturers wish to use other alarms and claim compliance, the procedure for doing so is transparent.

### Recommendations

The recommendation for manufacturers is to move quickly to using the auditory icon alarms rather than making adaptations to tonal alarms, which only became commonly used in medical devices for three reasons, two of which largely no longer apply. Firstly, the standard recommended the use of tonal alarms exclusively, so manufacturers complied. Secondly, the technology available to reproduce alarm sounds was of poor and limited quality. Thirdly, users are conservative and expect alarms to sound a particular way. There is still a cultural problem in moving away from tonal alarms to acceptance of better alarms that sound very different from expectations. Many medical devices now possess music-quality speakers that can reproduce sound considerably better than was possible previously. Using music-quality loudspeakers introduces a listening world where people can listen to more natural sound, and not in a way dictated by design restrictions brought about by unsophisticated and out-of-date technology.^28^^,^^29^

## Authors’ contributions

Study design and conception: all authors

Data collection: NT, NM

Analysis and interpretation of data: all authors

Manuscript drafting and critical revision: all authors

Approval of the manuscript submitted: all authors

## Declaration of interests

JRE has acted in the past as a consultant for Masimo Inc, and led the research project which updated the alarm signals contained within the 2020 version of IEC 60601-1-8. She is currently the UK representative on the technical committee (TC39) charged with producing the fourth edition of IEC 60601-1-8. The other authors declare that they have no conflicts of interest.
